# Reassessing Established Assumptions of Dietary Habits in the USA in the Context of Migration and Acculturation: a Qualitative Study of Latino Immigrants

**DOI:** 10.1007/s40615-024-01967-5

**Published:** 2024-04-26

**Authors:** Taynara Formagini, Daphnee Rodriguez, Julie Dias, Joanna Veazey Brooks

**Affiliations:** 1 Department of Family Medicine, University of California San Diego, San Diego, CA, USA; 2 Department of Population Health, School of Medicine, University of Kansas, Kansas City, KS, USA; 3 Department of Medicine, University of Central Florida, Orlando, FL, USA; 4 Department of Biological Sciences, University of Central Florida, Orlando, USA; 5 University of Kansas Cancer Center, Kansas City, KS, USA; 6 Division of Palliative Medicine, School of Medicine, University of Kansas, Kansas City, KS, USA

**Keywords:** Dietary acculturation, Latino immigrants, Immigration and diet, Obesity, Perceptions of healthy eating

## Abstract

**Introduction:**

The growing prevalence of obesity in the USA disproportionately affects Latinos compared to non-Latino Whites. Immigration and acculturation have been associated with unhealthy dietary shifts among Latino immigrants, a phenomenon known as dietary acculturation. Emerging evidence points to a more nuanced relationship between dietary habits, immigration, and acculturation, highlighting the need for a more current comprehension of dietary acculturation.

**Objective:**

We explored how Latino immigrants’ experiences in migrating to the USA have affected their perceived dietary habits and their experiences of how supportive the USA is in establishing healthy practices compared to their native country.

**Methods:**

Employing a descriptive qualitative study design, we conducted semi-structured interviews with 19 Latinos who had participated in a lifestyle change program between 2016 and 2019. We used thematic analysis to analyze the data and report emerging themes.

**Results:**

Participants expressed divergent perceptions of their dietary habits post-immigration. Some affirmed prevailing assumptions of dietary acculturation, citing deteriorating diet quality in the USA in the context of a faster pace of life, healthier options in the native country, and shifts in the food environment that prevented access to healthy foods. Conversely, others held opposing views, attributing their perceived improved diet to unhealthy dietary habits in Latin America, coupled with increased access to and affordability of healthy foods in the USA.

**Conclusion:**

Our study contributes to the evolving understanding of dietary acculturation among Latino immigrants and provides a more nuanced and updated understanding of this process that reflects their current experiences in acculturating to the USA.

## Introduction

Latinos^1^ represent 18.7% of the US population [1]. By 2060, this number is projected to increase considerably, and this group will make up 27.5% of the country’s population [2]. Latinos face one of the highest rates of obesity in the USA, only being surpassed by non-Latino Blacks. A total of 45.6% of Latino adults have obesity, compared to 41.4% of non-Latino Whites [3]. Considering that obesity is a chronic condition and a significant risk factor for cardiovascular diseases [4], addressing the growing prevalence of obesity among this rapidly growing minority group matters for reducing health disparities and is imperative for the health of the nation.

A large body of research on obesity risk factors among Latinos focuses on uncovering how migration to the USA and adoption of the country’s mainstream culture shape Latinos’ health behaviors and outcomes [5, 6]. Due to the substantial link between diet and obesity, there is a strong emphasis on examining Latino immigrants’ dietary habits and behaviors. Dietary habits encompass individuals’ food behaviors and patterns of food consumption and are influenced by factors such as culture, education, socioeconomic background, and health status [7]. Research highlights that as foreign-born Latinos acculturate to the behaviors, culture, and norms of the USA, they undergo a process known as *dietary acculturation*, where they tend to adopt the dietary habits of the USA and gradually abandon the dietary habits from their native country [8]. In fact, longer time in the USA and higher acculturation levels have been generally associated with higher consumption of fats and sugary drinks, higher consumption of fast-food options, and lower intake of fruits and vegetables [6, 9–12]. A prevailing assumption in this body of research is that Latinos consistently have healthier dietary habits prior to living in the USA, with migration often considered as the presumed catalyst for adverse changes in their diets [5, 13].

While the concept of dietary acculturation is widely acknowledged, growing evidence points to a more nuanced and complex relationship between dietary habits, immigration, and acculturation [14]. First, due to the complexity of measuring acculturation and diet, studies use different measures, which often leads to non-homogeneous findings. In fact, some epidemiological data have pointed to a positive effect of dietary acculturation [15, 16]. Second, despite generally sharing a common language and some cultural attributes, Latino immigrants constitute a diverse group, with individuals stemming from different life experiences and resources prior to migration. Studies reveal that dietary habits in Latin American countries vary based on factors such as sex, age, and socioeconomic status, challenging the assumption that all Latino immigrants maintain healthier diet quality prior to migration [17–19]. For example, one large cohort study conducted in the USA found significant variation in dietary habits across Latinos from different heritage groups [9]. Third, globalization and urbanization, particularly in the last two decades, have led to a transition in dietary habits in Latin America, characterized by increased consumption of energy-dense and processed foods and decreased consumption of fruits, vegetables, and whole grains [19–22]. In fact, obesity rates are increasing in Latin America, and Mexico has one of the most alarming rates of obesity in the continent [23, 24]. Lastly, studies emphasize how social and structural factors, such as socioeconomic status, housing, working conditions, and discrimination, play a key role in shaping the diet of Latinos as they integrate into US society [11, 14]. Taken together, these factors underscore the complexity of dietary acculturation, emphasizing an ongoing need to understand how migration and acculturation are *currently* affecting Latino dietary habits in the context of a changing landscape.

Our study contributes to this changing body of research by providing an exploratory qualitative analysis of Latino immigrants’ own perceptions of their dietary habits in the USA. Through semi-structured interviews, we explored how Latino immigrants’ experiences in migrating to the USA affected their perceptions of dietary habits and their experiences of how supportive the country is in establishing healthy habits compared to their native country. An updated understanding that reflects the current realities of how Latinos perceive their dietary habits within the context of immigration and acculturation can potentially inform larger studies analyzing diet quality before and after immigration and support targeted interventions to improve diet quality among this group.

## Methods

### Design and Setting

This study was part of a larger qualitative project exploring diabetes prevention behaviors among Latinos who participated in a lifestyle change program. Participants were interviewed a minimum of 12 months after participating in the program, with cohorts ranging from 2016/17 to 2018/19. Semi-structured interviews were conducted March–August 2021. To recruit participants, we partnered with a center that delivers a 12-month culturally-adapted lifestyle change program to Spanish-speaking Latinos. The program aimed to motivate participants to adopt dietary and physical activity modifications for weight loss. Following the Spanish version of the CDC PreventT2 curriculum, the program covered various topics, including strategies for becoming more active, tracking physical activity and food intake, making healthy food choices, navigating shopping and cooking, managing stress, coping with triggers, and sustaining motivation. Additional information on the PreventT2 curriculum can be accessed elsewhere [25].

In this manuscript, we report the data from participants’ perceptions of their dietary habits in regard to culture, acculturation, and the process of living in the USA. Prompts in this section of the interview did not include experiences related to their participation in the lifestyle change program. We followed the Standard for Reporting Qualitative Research (SRQR) [26] to ensure transparency in reporting the methods and findings of this study. The project was approved by the University of Kansas School of Medicine institutional review board [00146443].

### Participants and Data Collection

**Fig. 1:**
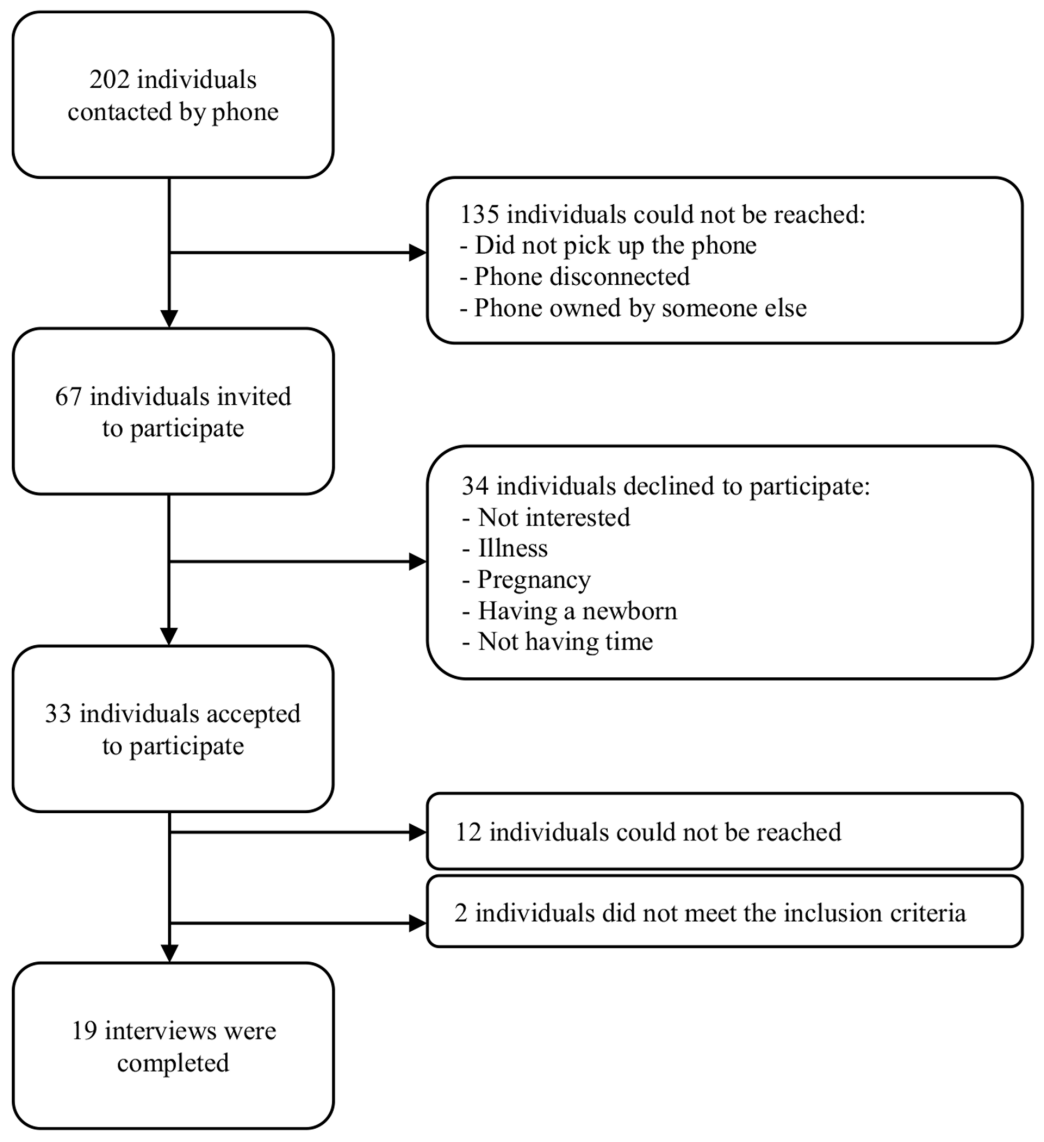
Flowchart

Theoretical purposive sampling [27] was used to recruit individuals who participated in the lifestyle change program. Additional inclusion criteria included being able to speak/read in English or Spanish and the ability to provide verbal consent. A Spanish-speaking community health worker who worked at the center contacted individuals by phone to invite them to participate in the interview and explain the study’s main objectives. When individuals demonstrated interest, an interview with the first author (who is Latina and fluent in Spanish) was scheduled at a time convenient for the participant. A total of 202 persons were initially contacted, of which 135 could not be reached after a maximum of three attempts. Detailed information about our recruitment process is illustrated in Fig. 1.

Data collection was terminated once all potential participants had been contacted or attempted to be contacted. Furthermore, we chose to stop attempting to recruit additional participants during preliminary analysis when investigators concurred that code saturation had been reached (i.e., no new themes were emerging from the interviews) [28]. A total of 19 interviews were completed, 17 by phone and two by videoconference. Interviews were audio-recorded, and all participants consented to participate verbally. Participants received a $25 gift card to compensate for their time.

### Research Materials

All research materials used in this study were created to be at an approximate 5th-grade reading level, verified by an online grade-level readability tool [29]. The materials were initially developed in English and then translated into Spanish by the first author. To ensure the materials were both culturally and linguistically appropriate, two native Spanish speakers not involved in the study reviewed the documents, focusing on verifying that the ideas captured the intended meaning. The interview guide was developed based on an extensive literature review, and it prompted participants to discuss (1) whether and how they perceived changes in their dietary habits in the process of moving and living in the USA and (2) their perceptions of the US food environment compared to their native country (see Supplementary Material).

### Sociodemographic Questionnaire

Participants answered a brief questionnaire containing information about their sex, age, marital status, country of birth, time in the USA in years, educational level, income, work status, family size, health insurance, and language spoken at home. To capture participants’ acculturation, we computed an acculturation score using three variables obtained from the sociodemographic questionnaire: country of birth, language spoken at home, and time in the USA. These three variables are proxies for acculturation commonly reported in prior research, and this method of calculating a score based on these variables aligns with previously published studies on acculturation and Latino health [30, 31]. Each variable was assigned a binary point (0 or 1) based on the following criteria: US-born (1 point) vs. foreign-born (0 points); English-speaking (1 point) vs. Spanish-speaking (0 points); and living in the USA for 10 years or more (1 point) vs. less than 10 years (0 points). We summed the scores for each participant, creating an acculturation score that ranged from 0 (indicating the lowest acculturation level) to 3 (indicating the highest acculturation level).

### Data Analysis

The interviews were transcribed in Spanish via a web-based transcription platform. The first author checked the transcripts for accuracy, translated the transcripts into English, and deidentified the data. Verbatim translations were not used, but rather, the content was translated with the aim of maintaining the original meaning expressed by participants. In addition, anytime questions arose regarding the meaning of the quotes, the original Spanish transcriptions were checked, and the quotes were reviewed whenever necessary. The data was coded using the NVivo 12 qualitative software [32].

We used thematic analysis to analyze the data, which aims to identify, analyze, and report patterns from the dataset by creating themes from the patterns found [33, 34]. Following the steps of thematic analysis, two investigators [TF and JB] initially coded a subset of four interviews separately. This process consisted of taking first-impression notes, observations, and interpretations and identifying any emerging patterns and overall themes. Afterward, the two investigators met to discuss the patterns found and solve any discordances. An initial codebook was developed, which included the names of these initial patterns or codes and their description and examples. The codebook was used to code the remaining transcripts, allowing for new themes to emerge from the data. We then conducted a review of the codebook to change codes’ names and combine less prominent codes whenever needed. In this manuscript, we focused on reporting the findings from the code “diet: native country vs. USA.” This code included all interviews’ content related to their experiences with dietary acculturation. We organized the findings by the most significant themes. To preserve anonymity, the quotes from the participants are presented with a randomly assigned identifier, along with the participant’s sex, age, years in the USA, and acculturation score (AS).

## Results

### Participants’ Sociodemographic Characteristics and Acculturation Level

Table 1 reports participants’ sociodemographic characteristics and acculturation levels. Nearly all participants were female (*n* = 16, 84.2%), with a mean age of 41 years old. A total of 17 participants (89.5%) were born in Mexico, and 15 have been living in the USA for 10 years or more (78.9%). Most of the participants did not complete high school education (*n* = 12, 63.1%). Furthermore, participants had overall low income, with 10 reporting total household income between $20k and $39k (52.7%). Similarly, 10 participants reported having an income that was 130% under the Federal Poverty Level (52.6%). A total of 16 participants spoke only Spanish or more Spanish than English at home (84.2%). Most participants had relatively low levels of acculturation. Mean acculturation was 1.3, with 3 participants (15.8%) scoring zero, 9 (47.3%) scoring one, 6 (31.6%) scoring two, and only 1 (5.3%) scoring three points in the AS.

### Perceptions and Experiences of Dietary Habits in the USA

Data from participants’ interviews revealed two main themes regarding perceptions of dietary habits in the context of immigration and acculturation to the USA: (1) Consistent with conventional assumptions of dietary acculturation and unhealthy dietary shifts, some participants shared experiences that described potential negative dietary practices as a result of migrating to the USA. (2) In contrast, others presented experiences that are consistent with the growing evidence of a more nuanced and complex relationship between immigration to the USA and dietary habits. Notably, this group highlighted perceptions of unhealthy food practices prior to migration, potential healthy dietary habits in the USA, and increased access and affordability of healthy foods in the USA. We present both themes below, along with illustrative quotes; additional quotes are included in Table 2. We first report the data consistent with established assumptions of dietary acculturation.

1) Conventional Narratives: Unhealthy Dietary Shifts

Three sub-themes that provide the context for experiences of unhealthy dietary shifts were discussed by participants: faster pace of life, healthier options in the native country, and shifts in the food environment.

#### Faster Pace of Life

In line with prior qualitative evidence linking dietary habits to challenging working conditions [24, 35, 36], some participants expressed they changed their diet as a result of the adjustments to a new life in the country, mostly related to work conditions that made it difficult to maintain the same diet as they used to have in their native country. These narratives centered on the lack of time to prepare food at home, leading to the purchase of ready-to-eat foods.

… *we work all day. You get tired, and you don’t want to prepare something healthy if you don’t buy something fast. That would be an obstacle, first getting tired of working and cooking something healthy. It’s easier for us to buy something… fast food*. [12] [39, female, ≥10 years, AS=1]

#### Healthier Options in the Native Country

It was common for participants to discuss that their native country had a higher availability of healthier food options compared to the USA, including more fresh food and vegetables and lower availability of meat in meals. Experiences of not finding the same types of foods they used to eat in their native country were also considered.

*Well, In Mexico […] I ate more vegetables, I almost didn’t eat much meat. And here, yes. When I got here, at first it affected me a lot in my stomach, because here everyone eats meat [laughs]*… [78] [34, female, <10 years, AS = 0]

#### Shifts in the Food Environment

In addition, some participants shared stories about the shifts in the food environment, contrasting what they were exposed to in their native country to the USA. They highlighted their past ability to harvest their own organic foods, including vegetables, eggs, and milk.

*We eat healthier [in Mexico], more organic, because there you go to the field and cut, for example, the cactus, which we call nopales. We cut some herbs that are called quelites. A few verdolagas, some mushrooms. We eat vegetables without chemicals and nothing. Everything is more organic*. [60] [28, female, <10 years, AS = 0]

Participant 32 mentioned the absence of a popular fast-food chain in her hometown in her native country, which she believed used to limit access to such foods and encouraged homemade meal preparation.

*Well, as an example. if I want[ed] to eat at McDonald’s. I have to go for an hour on a bus to buy a McDonald’s and some French fries. Then better to just stay at the house. I [could] prepare something to substitute it, […] instead of eating something that’s going to be bad*. [32] [42, female, <10 years, AS=0]

2) Reassessing Assumptions: Nuanced Diet Experiences

On the other hand, some participants shared experiences that were consistent with the growing evidence of a more nuanced and complex relationship between immigration to the USA and dietary habits. Three sub-themes emerged from the interviews, including perceptions of unhealthy dietary habits in Latin America, healthier dietary habits in the USA, and improved access and affordability in the USA to purchase healthier foods.

#### Unhealthy Dietary Habits in Latin America

In reflecting on the dietary habits in Latin America, some participants expressed that, in their view, their native country did not foster healthy dietary habits. These participants, all of Mexican heritage, highlighted foods like quesadillas, tacos, and potatoes, along with the traditional preparation method—frying of popular dishes—as integral to the cultural norms of Mexico.

*We Mexicans, we like, for example, quesadilla, meat with potatoes. Well, this is not, it’s not healthy. We barely put vegetables in it, which is more important than fruit.[…] We are very used to rice, beans, potatoes. It’s like part of our culture. The corn, […], all of these have carbohydrates. […] The tacos are fried, the enchiladas are fried. Yes, it’s a lot… I think it has a lot to do with the way of cooking Mexican food*. [45].[38, female, ≥10 years, AS = 1]

One participant directly mentioned that there was a cultural shift in Mexico from healthier practices in the past to a current decline in health consciousness, which, according to him, is impacting food practices in the country. Here, while this does not imply that he had unhealthier dietary habits, it suggests a perceived deterioration in diets in Mexico.

*A few years ago, when I lived there […] people did not have information, but it was the culture, the customs. I think we used to eat healthier. I remember my childhood […] growing up we ate mostly healthy things, we ate a lot of vegetables produced by the countryside […] But today I have realized that all those things have changed and then nowadays people take less care of their health*. [85] [58, male, ≥10 years, AS = 1]

#### Healthier Dietary Habits in the USA

Participants expressed a perception of a positive shift in their dietary habits as a result of living in the USA, with an emphasis on incorporating salads and fruits into their diet. One participant described salads and fruits as “American foods.” These narratives collectively highlight a potential positive shift toward incorporating healthier food choices within the USA food environment as perceived by the participants.

*Ah, nowadays I have learned to eat more American food because I, for example, in Mexico, I didn’t eat much, didn’t eat much salad, and I learned to eat them here. Here is where I learned to eat more, more salads and more fruit*. [35].[57, female, ≥10 years, AS = 1]

#### Improved Access and Affordability in the USA

Finally, certain participants emphasized improved opportunities to access healthier food options in the USA. The discussion included considerations of the affordability of healthy foods, particularly fruits and vegetables, and alternatives like chicken and fish compared to red meat.

*The diets are… They may be better in the United States because there are more, more chances of buying healthy things here […] It is more available […] Well, it is cheaper and everything? You know? And you can buy it*. [68]. [43, male, ≥10 years, AS=1]

## Discussion

This study used semi-structured interviews to explore the experiences of Latinos in the context of migration and acculturation to the USA in connection with the perceived healthfulness of their diet. Our study provides some support to current assumptions of dietary acculturation and the deterioration of Latino immigrants’ dietary habits as a consequence of migrating to the USA. Notably, we also found evidence that dietary acculturation is nuanced and complex and might signify perceived healthier dietary habits in the USA, or a continuation of unhealthy dietary habits carrying on from practices acquired prior to migration. This range of responses and experiences potentially suggests a transition in dietary habits in Latin America due to globalization and urbanization and highlights the heterogeneity within the Latino population, potentially reflecting a range of dietary habits both in their native country and in the USA. Our study contributes to the growing body of work that examines the complexity of migration and dietary acculturation by reassessing established assumptions of expected unhealthy dietary shifts, based on the perception of Latino Immigrants [14]. An updated understanding of the dietary journey in the process of immigration and acculturation may be useful to inform future studies that assess dietary habits and behaviors among Latino immigrants in the USA.

A large body of epidemiologic research suggests that longer time in the USA and higher acculturation levels are associated with poorer diet quality, which includes a decreased consumption of fruits and vegetables and increased consumption of fast foods and sugary drinks [37–40]. These studies indicate that Latino immigrants’ dietary habits become increasingly worse as they acculturate to the USA, thus increasing their risk of developing obesity and other chronic and cardiovascular diseases [41, 42]. In line with these prior investigations, our analyses indicated that, as narrated by certain participants, changes in dietary habits occurred as part of their immigration journey. In our study, consumption of an unhealthy diet was perceived to be associated with the faster pace of life in the USA, the availability of healthier food options in the native country, and the different food environment in the USA, which was considered to be less supportive of healthy dietary habits.

Structural barriers, including inadequate housing, demanding work schedules, and other economic constraints, can limit access to healthy food options among Latinos in the USA. In fact, the association between poor living conditions and low diet quality among Latino immigrants has been consistently presented in previous research [5, 43–45]. In our study, participants attributed unhealthy dietary shifts to time constraints from work, which prevented home cooking. Other qualitative studies have also found that busy schedules and time pressures were reported by Latinos as reasons for changing their dietary routines and purchasing more fast food [35, 36]. For example, Latinas from Honduras reported that their dietary habits were impacted by their busy schedules and limited time to cook in the USA, leading to increased consumption of more affordable and convenient fast foods [35]. Sussner and colleagues (2008) found that immigrant Latina mothers attributed their poor dietary habits to a lack of personal time, competing demands of work, and busy lives in the USA [36].

Similar to previous reports [16, 37, 46], some participants in our study stated that they consumed more vegetables and fruits in their native country, and in contrast, they now consume more fast foods, meat, and soda due to the higher availability of these types of foods in the country. Experiences of increased consumption of calorie-dense and nutrient-poor foods post-immigration support established claims of dietary acculturation [40]. In a systematic review that included five qualitative studies, Ayala and colleagues (2008) showed that fresh foods were perceived by Latino immigrants as more readily available in their native countries. Yet, the authors discussed that this availability appears to vary by country of origin, with Honduran women reporting that they had little access to fresh vegetables prior to migration [16].

In accordance with the growing evidence suggesting a more nuanced and complex relationship between dietary habits, immigration, and acculturation, our findings provide data on the heterogeneity of participants’ experiences regarding dietary habits prior to and after migration to the USA. Participants pointed to the unhealthy nature of foods from their native country, healthier diets in the USA, and increased affordability of healthy food options in the country. Two other qualitative studies report similar findings: in one study, participants perceived foods from Mexico to be unhealthy, and many described salad and grilled chicken as “healthier” and “American foods” [47]. In another study, participants revealed they engaged in negative dietary practices prior to immigration, including consuming food outside of the home and integrating processed food into their cooking [13]. Likewise, two studies employing US nationally representative data demonstrated that increased acculturation levels were linked to greater whole grain intake among Mexican Americans [40, 48], even amid declining diet quality in other dimensions, which highlights the complexity of diet formation as immigrants assimilate to the USA.

Our participants also discussed that dietary habits in their native country were not necessarily healthier than the practices in the USA, providing potential evidence for the research highlighting the changes in Latin America’s food environment due to globalization and urbanization in the past two decades. Studies have, in fact, shown that Latin American countries have high consumption of foods high in fats and sugar and highly processed foods, along with a suboptimal intake of fruits and vegetables [17, 49, 50]. These findings may be explained by the nutrition transition or Westernization theory, which indicates that as countries shift to be more similar to higher-income countries, such as the USA, the patterns of diets change. Diets typically become less nutritious, marked by lower consumption of fibers and increased consumption of fats and animal proteins [51–53].

Our data also further suggests that some Latino immigrants perceived they adopted healthier dietary habits in the USA due to the increased affordability of nutritious foods compared to their native country. This observation aligns with the recognized heterogeneity within the Latino community, potentially reflecting individual experiences of upward mobility post-migration, which could facilitate improved access to healthy foods. In a recent publication using nationally representative data, Ramirez and colleagues (2022) showed that Latinos with higher income and lower acculturation had higher diet quality compared to those with lower income and higher acculturation, driven by increased consumption of greens, beans, and whole grains [11]. Notably, our participants generally had low socioeconomic status, but it is possible they faced even greater constraints accessing healthy foods in their native country.

Of note, several limitations are associated with this study. First, participants’ perceptions of their dietary habits might not directly reflect their actual dietary behaviors, and their understanding of what constitutes a healthy diet might not consistently align with nutritional norms or guidelines. The occurrence of this second scenario is less probable, given that all participants had previously been involved in a lifestyle change program, and previous findings suggest the program was helpful in improving participants’ health literacy as related to dietary habits [54]. Second, because we recruited Latinos who have participated in a lifestyle change program, their perceptions of the USA being more or less supportive of a healthy diet could be a result of the knowledge acquired in the program or the potential impact of social desirability. Third, our sample does not represent all Latinos living in the USA, particularly considering that most participants were females, of Mexican origin, and generally with low acculturation levels. Moreover, participants’ recall of their dietary practices before immigration might be subject to memory biases. Bicultural individuals might associate unhealthy special occasion foods with their native country despite not being eaten on a regular basis, a phenomenon called the “festival foods hypothesis” [55]. Finally, the homogeneity of participants’ acculturation level limited our ability to discern any potential patterns among participants’ experiences. Future studies can assess whether and how perceptions of dietary habits among this group vary by acculturation level or time in the USA. Despite these limitations, our study captures a broad spectrum of views on dietary habits in the context of immigration and acculturation, including a range of participants with varying backgrounds and experiences, such as age, time in the USA, socioeconomic status, and acculturation levels.

## Conclusions and Implications

Our study contributes to the evolving understanding of dietary acculturation among Latino immigrants in the USA and provides a more nuanced and updated understanding of this process that reflects their current experiences in acculturating to the country. The perspectives described in our study offer insights into potential mechanisms through which dietary practices undergo positive and negative changes. Future studies should compare nutritional availability, access, and intake between Latin American countries and the United States. Studies can also explore pre-post changes in participants’ perceptions of dietary habits resulting from participation in lifestyle change programs. Prospective cohort studies can follow the diet of Latinos in the USA starting early upon arrival to provide a more comprehensive understanding of the short and long-term changes associated with integration and acculturation to US society. Future research should investigate how one’s perceived healthfulness of their diet aligns with or deviates from dietary guidelines. Larger qualitative studies could compare different Latino sub-groups, particularly by country of origin, to uncover potential differences in dietary acculturation experiences. Understanding how socioeconomic integration into the USA currently affects dietary practices and dietary acculturation might be important as well. Finally, future behavioral programs aimed at promoting improved diet quality among Latino immigrants can use this information in the design of interventions by highlighting instances where dietary acculturation might lead to healthier practices. By incorporating a more nuanced and updated understanding of dietary acculturation, public health research and practice might provide targeted strategies for promoting healthier practices among Latinos in the USA.

## Supplementary Material

supplementary material

## Figures and Tables

**Table 1:** Participants’ characteristics (*N* = 19)

Characteristic	Distribution
Female, n (%)	16 (84.2)
Age, mean (SD)	41 (8.5)
Marital status, n (%)	
Married/Cohabiting	17 (89.5)
Divorced/Separated	2 (10.5)
Country of birth, n (%)	
Mexico	17 (89.5)
El Salvador	1 (5.3)
Guatemala	1 (5.3)
Time in the USA, n (%)	
Less than 10 years	4 (21.1)
10 years or more	15 (78.9)
Education level, n (%)	
Less than high school	12 (63.2)
High school or higher	7 (36.8)
Household income, n (%)	
Less than $20,000	2 (10.5)
$20,000–$29,999	6 (31.6)
$30,000–$39,999	4 (21.1)
$40,000–$49,999	3 (15.8)
$50,000 or more	3 (15.8)
Unsure	1 (5.3)
Household family size, mean (SD)	3.89 (1.2)
Income relative to the Federal Poverty Level, n (%)^a^	
Under 130%	10 (52.6)
130% or above	8 (42.1)
Unsure	1 (5.3)
Work status, n (%)	
Employed full-time	7 (36.8)
Employed part-time	5 (26.3)
Self-employed	1 (5.3)
Unemployed/Housemaker	6 (31.6)
Covered by health insurance, n (%)	2 (10.5)
Language spoken at home, n (%)	
Only Spanish	8 (42.1)
More Spanish than English	8 (42.1)
English and Spanish equally	3 (15.8)
Acculturation score *(AS)*	
Mean (SD)	1.3 (0.8)
Score = 0 (lowest), n (%)	3 (15.8)
Score = 1, n (%)	9 (47.3)
Score = 2, n (%)	6 (31.6)
Score = 3 (highest), n (%)	1 (5.3)

a Calculated based on household income and household family size

**Table 2:** Themes, sub-themes, and supporting participants’ quotes

Theme 1. Conventional Narratives: Unhealthy Dietary Shifts	
Faster pace of life	*Because sometimes we leave work very late. So, we start [working] early, we leave [work] late, then we get to the house, and we don’t have much time left to cook and all that*. [66] [38, female, ≥ 10 years, AS = 2] *The life here [in the U.S.] is very different than in Mexico. I mean, there are women who have to work, […], men who have to work […] they live a very fast, very, very hard life. And it is not easy, and they buy frozen food or buy food on the street and do not cook at home. […]. One of the problems coming to the United States is that people hardly have time […] Because of work.* [73] [37, female, ≥ 10 years, AS = 2]
Healthier options in the native country	*I think more [healthy in Mexico], because we also have a lot of food that doesn’t necessarily have to have meat. Maybe it can have fat, but instead of drinking soda, we drink cucumber water, water, and lemon. […] I make [my family] fruit water instead of juice if they want to drink something sweet* [42]. *[30, female*, ≥ *10 years, AS* = *2]* *Oh, so [my diet] changed in matters of, you don’t find all the vegetables you’re used [to eat] from Mexico. [66] [38, female*, ≥ *10 years, AS* = *2]* *[…] And meat, we only ate meat on some special occasions. [60] [28, female*, < *10 years, AS* = *0]*
Shifts in the food environment	*We [now] eat tortillas in the morning and then fried eggs, chorizo, even soda in the morning. And that’s a lot of calories for a single meal. […] There [in Mexico] everything is more fresh. Because eggs are caught from the chicken, and the cheese comes from the cow, the milk you drink was also taken out of the cow. And here we drink milk that comes in a bottle.[93] [35, female*, ≥ *10 years, AS* = *2]*
Theme 2. Reassessing Assumptions: Nuanced Diet Experiences	
Unhealthy dietary habits in Latin America	*I think Mexican culture grew based on carbohydrates. And it’s because you eat a lot of bread, a lot of tortillas… My uncles and aunts, who are there, for all meals, always have soda. It’s something I look at it in some Mexican families here. But in American families, soda is almost not used. So, I think…, Well, the Mexican culture it’s more about not eating healthy* [48]. *[47, female*, ≥ *10 years, AS* = *2]* *Normally… we are Mexicans. Our traditional foods are not healthy. So that’s where you don’t eat healthy food.* [34]. *[42, female*, ≥ *10 years, AS* = *1]* *The way I see it, well, here in the United States, it’s not so much… Healthy eating, it’s just more junk food. And Mexico. Yes, yes, there are vegetables and all that, but we also cook very greasy [food] too* [12]. *[39, female*, ≥ *10 years, AS* = *1]*
Healthier dietary habits in the USA	*…I eat a lot of salads. I didn’t eat like that in my country. I do think it’s more supportive of healthy food here [in the U.S.] [98]. [41, male*, ≥ *10 years, AS* = *3]* *… here in the United States, in general, there are very delicious salads. In the same way, in Mexico, we have various types of food with lots of vegetables. I would say is each person’s choice on how much they eat* [37]. *[37, female*, ≥ *10 years, AS* = *2]*
Improved access and affordability in the USA	*There are places [here] where you can get quality food at a good price. […] For example, the fruit and vegetables that we should eat are cheaper than meat.* [45]. *[38, female*, ≥ *10 years, AS* = *1]* *Yes, because I also saw the change in my income […] nowadays I buy more with less money […] I used to [eat] a lot of red meat. And red meat is more expensive than chicken, and [chicken] is healthier. And it’s cheaper, more economical. And fish, which is also a little cheaper than meat, because meat is more expensive.* [35]. *[57, female*, ≥ *10 years, AS* = *1]*

## Data Availability

The authors welcome reasonable requests for non-identifiable data and materials and will carefully consider these requests.

## Footnotes

1 We used the term Latinos interchangeably with Hispanics, Latinas, or Latinxs.
