# Supplementary material for: Reassessing Established Assumptions of Dietary Habits in the USA in the Context of Migration and Acculturation: a Qualitative Study of Latino Immigrants
Source: J Racial Ethn Health Disparities. Author manuscript; Available in PMC 2025 Apr 1. (PMC11914326; doi:10.1007/s40615-024-01967-5)
Supplement: supplementary material [file NIHMS2061806-supplement-supplementary_material.docx]

**Reassessing established assumptions of dietary habits in the U.S. in the context of migration and acculturation: a qualitative study of Latino immigrants**

### Supplementary Material: Section of the Interview guide

**Prompts related to dietary habits in the context of acculturation and immigration to the U.S.**

1. Do you think the type of food you eat has anything to do with your culture?
2. Do you eat more North American food or food from your country of origin? Tell us more about that.
3. Do you think the culture where you grew up was more or less supportive of a healthy diet compared to the U.S. culture? Why or why not?
4. Did you change your diet or the foods you routinely eat once you came to the U.S.? How?
